# Obesity and critical care nutrition: current practice gaps and directions for future research

**DOI:** 10.1186/s13054-022-04148-0

**Published:** 2022-09-20

**Authors:** Roland N. Dickerson, Laura Andromalos, J. Christian Brown, Maria Isabel T. D. Correia, Wanda Pritts, Emma J. Ridley, Katie N. Robinson, Martin D. Rosenthal, Arthur R. H. van Zanten

**Affiliations:** 1 Department of Clinical Pharmacy and Translational Science, University of Tennessee Health Science Center, 881 Madison Avenue, Suite 345, Memphis, TN 38163 USA; 2 Clinical Nutrition Manager, Sodexo, Minneapolis, MN USA; 3 Digestive Disease and Surgery Institute, Cleveland Clinic, Cleveland, OH USA; 4 Department of Surgery, Federal University of Minas Gerais, Belo Horizonte, Brazil; 5 Placenta-Linda Hospital, 1301 Rose Dr, Placentia, CA USA; 6 Australian and New Zealand Intensive Care Research Centre, School of Public Health and Preventive Medicine, Monash University, Melbourne, VIC Australia; 7 Scientific and Medical Affairs, Abbott Nutrition, 2900 Easton Square Pl, Columbus, OH USA; 8 Acute Care Surgery Team, University of Florida College of Medicine, Gainesville, FL USA; 9 Chair, Department of Intensive Care Medicine & Research, Gelderse Vallei Hospital, Willy Brandtlaan 10, 6716 RP Ede, The Netherlands; 10 Division of Human Nutrition and Health, Chair Group Nutritional Biology, Wageningen University & Research, HELIX (Building 124), Stippeneng 4, 6708 WE Wageningen, The Netherlands

**Keywords:** Malnutrition, Obesity, Critical illness, Nutrition assessment, Nutritional requirements, Nutrition therapy

## Abstract

**Background:**

This review has been developed following a panel discussion with an international group of experts in the care of patients with obesity in the critical care setting and focuses on current best practices in malnutrition screening and assessment, estimation of energy needs for patients with obesity, the risks and management of sarcopenic obesity, the value of tailored nutrition recommendations, and the emerging role of immunonutrition. Patients admitted to the intensive care unit (ICU) increasingly present with overweight and obesity that require individualized nutrition considerations due to underlying comorbidities, immunological factors such as inflammation, and changes in energy expenditure and other aspects of metabolism. While research continues to accumulate, important knowledge gaps persist in recognizing and managing the complex nutritional needs in ICU patients with obesity. Available malnutrition screening and assessment tools are limited in patients with obesity due to a lack of validation and heterogeneous factors impacting nutrition status in this population. Estimations of energy and protein demands are also complex in patients with obesity and may include estimations based upon ideal, actual, or adjusted body weight. Evidence is still sparse on the role of immunonutrition in patients with obesity, but the presence of inflammation that impacts immune function may suggest a role for these nutrients in hemodynamically stable ICU patients. Educational efforts are needed for all clinicians who care for complex cases of critically ill patients with obesity, with a focus on strategies for optimal nutrition and the consideration of issues such as weight stigma and bias impacting the delivery of care.

**Conclusions:**

Current nutritional strategies for these patients should be undertaken with a focus on individualized care that considers the whole person, including the possibility of preexisting comorbidities, altered metabolism, and chronic stigma, which may impact the provision of nutritional care. Additional research should focus on the applicability of current guidelines and evidence for nutrition therapy in populations with obesity, especially in the setting of critical illness.

## Introduction

Patients admitted to the intensive care unit (ICU) increasingly present with obesity, with rates reported between 28.2% and 36% [1–3]. In 2000, the World Health Organization (WHO) recognized obesity (BMI ≥ 30 kg/m^2^) as a distinct disease, describing the condition as a global pandemic with the potential to surpass more traditional world health problems, such as undernutrition and infectious diseases [4]. Since 2000, multiple societies [5–9] have all affirmed the classification of obesity as a disease. The WHO defines obesity as a condition in which excess or abnormal body fat accumulation increases health risks [4, 10]. Specifically, overweight and obesity are important contributors to morbidity and mortality due to increased risks of hypertension, dyslipidemia, type 2 diabetes, coronary heart disease, stroke, certain types of cancer, and other chronic conditions [4]. The Obesity Society issued a statement emphasizing the definition of obesity as a multicausal, chronic disease associated with structural abnormalities, physiologic derangements, and functional impairment accompanied by an increased risk of morbidity and early mortality [6]. Furthermore, nutritional deficiencies and malnutrition are likely underdiagnosed in patients with overweight and obesity related to the lack of highly sensitive and specific assessment tools and established diagnostic criteria.

The ongoing COVID-19 pandemic provides a unique lens through which to view obesity risks to the overall population and the complex presentation and clinical needs of patients with obesity [11]. Obesity is common in patients hospitalized with COVID-19. Of more than 148,000 patients who received a diagnosis of COVID-19 in US emergency departments or inpatient units between March and December 2020, 50.8% had obesity [12]. A meta-analysis of available data from January 1, 2020, to August 11, 2020, found that pooled mortality rates among patients hospitalized with COVID-19 were approaching 19%, and obesity was identified as a significant risk factor for mortality for these patients [13]. Meanwhile, the pandemic has not only negatively impacted the care of hospitalized patients with obesity but may also be contributing to population-wide weight gain [14]. In patients with preexisting obesity, the COVID-19 pandemic has been associated with increasingly unhealthy eating patterns [15]. Even in those without preexisting overweight and obesity, issues resulting from self-quarantine such as inadequate sleep, snacking, lack of dietary restraint, stress-related eating, and reduced physical activity may be contributing to weight gain [16, 17].

Traditional diagnostic assessment tools like body mass index (BMI) may fail to exhibit linear patterns between individual BMI determinations and cardiometabolic health status [18] or associated risk in critical illness [19]. Although controversial, current research describes a J-shaped relationship between BMI and mortality risk in critical illness, suggesting that patients who are overweight or who have moderate obesity have a lower mortality risk than those with a normal range BMI or severe obesity [20, 21]. These findings, termed the obesity paradox, may be subject to selection bias related to the possibility that some of these individuals may have had a better nutrition status than those with a low BMI [22]. The data suggest that more precise measures of body composition and nutrition status may offer greater insight when determining individual morbidity risks, especially during critical illness.

**Fig. 1:**
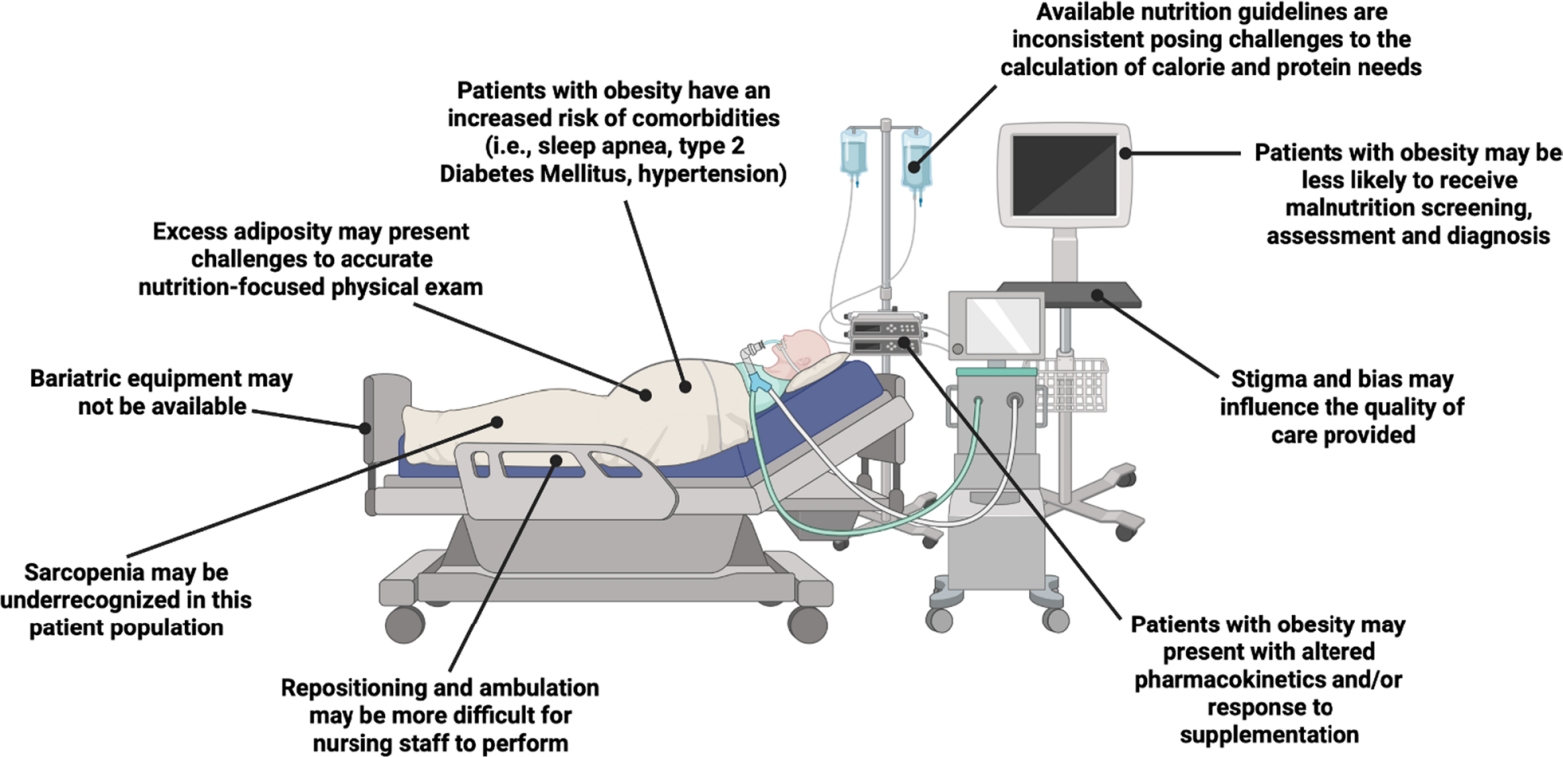
Factors complicating the care of critically ill patients with obesity. Created with BioRender.com

Guidelines are available for the nutrition care of critically ill patients with obesity, but underlying research in this area is limited. Societies such as the American Society for Parenteral and Enteral Nutrition (ASPEN), the Society of Critical Care Medicine (SCCM), and the European Society for Clinical Nutrition and Metabolism (ESPEN) have published recommendations for this population and often recommend an individualized approach to most aspects of nutritional care in patients with obesity (Table 1) [23–25]. A recently published update to the 2016 ASPEN/SCCM guidelines evaluated five questions central to nutritional therapy for all critical care patients. The guidelines did not specify recommendations for patients with obesity and instead, concluded that clinical judgment and close monitoring continue to be needed in the absence of consistent evidence [26]. Practitioners should also consider that a variety of factors can complicate the delivery of optimal nutritional care to patients with obesity. These patients may present with altered nutrient processing and pharmacokinetics, especially in the critical care setting, which potentially complicates both medication delivery and nutritional management efforts [26–32]. A summary of potential factors complicating the care of patients with obesity in critical illness is illustrated in Fig. 1.

**Table 1:** A review of guidelines and other consensus recommendations for nutritional management of patients with obesity in critical care

Clinical area	Guideline recommendations	Ongoing challenges	Consensus recommendations
Performing malnutrition screening and assessment	**ESPEN, ASPEN/SCCM:** A general clinical assessment should be performed to assess for malnutrition in the ICU, until a specific tool is validated [23, 24]	Current tools are not validated for patients with obesity and may rely on imprecise measures, such as BMI	Assessment of patients with obesity should consider the possibility of underlying malnutrition, based on clinical reasoning, signs and symptoms of macro/micronutrient deficiencies, and poor muscle quality despite the presence of muscle mass
	**ASPEN/SCCM:** Assessment in patients with obesity should also include biomarkers of metabolic syndrome, comorbidities, and inflammation markers [24]	Malnutrition may be underrecognized in patients with obesity	Monitor patients with obesity for refeeding syndrome over the course of treatment
Estimating energy needs	**ESPEN:** In patients with obesity, energy intake should be guided by IC. If IC is unavailable, energy intake can be based on adjusted body weight (actual body weight − ideal body weight) × 0.25 + ideal body weight) [23]	There is no consensus on how to estimate energy needs for critical care patients with obesity (ideal, actual, vs adjusted body weight)	IC is preferred to estimate energy needs among those with obesity in the ICU
	**ASPEN/SCCM:** IC should be used when available/feasible; otherwise use a published predictive equation or simple weight-based equation: 11–14 kcal/kg *actual body weight* per day for patients with BMI in the range of 30–50 and 22–25 kcal/kg *ideal body weight* per day for patients with BMI > 50 [24].	Predictive equations are easily calculated but were developed in populations without obesity	Predictive or weight-based equations should be only one aspect of a nutrition assessment, especially for patients with obesity
			Review individual nutritional requirements and factors that influence energy needs on a regular basis to adjust intake
	**ASPEN:** If IC unavailable, use Penn State Eq. 2010 or modified Penn State Equation depending on patient age [25]		Manage patients with the intention of reducing net protein catabolism without concurrent feeding complications, worsening of physical function, or clinical outcomes when compared to withholding nutritional therapy
Estimating protein needs and recognizing and addressing risk of sarcopenic obesity	**ESPEN:** Protein delivery should be guided by urinary nitrogen losses or lean body mass determination (using CT or other tools); if urinary nitrogen losses or lean body mass determination are not available, protein intake can be 1.3 g/kg “adjusted body weight”/day [23]	Clinicians may be unaware of risk of muscle mass loss in patients with obesity	Protein needs may be higher in critically ill patients with obesity
	**ASPEN/SCCM:** 2.0 g/kg ideal body weight per day for patients with BMI of 30–39.9 and up to 2.5 g/kg ideal body weight per day for patients with BMI ≥ 40 [24]	Weight loss in critical illness may contribute to the loss of lean body mass rather than just fat mass	Individualized approach to nutritional and body composition assessments
	**ASPEN: “**High-protein feeding may be started with 1.2 g/kg actual weight or 2–2.5 g/kg ideal body weight, with adjustment of goal protein intake by the results of nitrogen balance studies” [25]	A 24-h urine collection for a nitrogen balance determination may not be practical or feasible for some institutions	Frequently reassess clinical status and nutritional needs
Choosing the ideal nutritional regimen	**ASPEN/SCCM:** Hypocaloric, high-protein regimen for patients with obesity; assess regularly for adequate protein intake [24]	Renal status and nitrogen balance should be carefully monitored with high-protein intake, especially for older patients or those with underlying kidney disease [25, 26]	Consider an individualized approach to nutritional management that achieves a higher protein intake without overfeeding
	**ASPEN** “A trial of hypocaloric, high-protein feeding is suggested in patients who do not have severe renal or hepatic dysfunction” [25]		Maintain an individualized approach that recognizes changing nutritional needs over the course of illness
Using IMN	**ASPEN/SCCM:** “While an exaggerated immune response in obese patients implicates potential benefit from immune-modulating formulas, lack of outcome data precludes a recommendation at this time” [24]	Obesity creates a pro-inflammatory environment. The ability of nutrition to modulate this inflammation in patients with obesity is unclear	It is unclear whether IMN would be beneficial for routine use among ICU patients with obesity. However, IMN is suggested for routine use in patients with TBI and in the surgical ICU. Additionally, IMN should be considered for patients with severe trauma [23]

*ASPEN* American Society for Parenteral and Enteral Nutrition, *BMI* body mass index, *CT* computed tomography, *ESPEN* European Society for Clinical Nutrition and Metabolism, *IC* indirect calorimetry, *ICU* intensive care unit, *IMN* immunonutrition, *SCCM* Society of Critical Care Medicine, *TBI* traumatic brain injury

While research continues to accumulate, important knowledge gaps persist in recognizing and managing the complex nutritional needs in ICU patients with obesity. This paper is the result of an international advisory board consisting of experts in obesity care in the ICU working to address these gaps from dietitian, physician, pharmacist, and nursing perspectives. The advisory board aimed to identify key knowledge gaps, challenges, clinical considerations, and outstanding questions when providing optimal nutritional care for critically ill patients with obesity. Consequently, this review will focus on current best practices in malnutrition screening and assessment, estimation of energy and protein needs, the risks and management of sarcopenic obesity, the value of tailored nutrition recommendations, and the emerging role of immunonutrition for patients with obesity.

## Malnutrition screening and assessment

Malnutrition screening and assessment is a key challenge in patients with overweight and obesity, and it may not be routinely performed. Thus, malnutrition as a diagnosis may be underestimated for these patients. Patients with malnutrition and obesity were less likely to have a malnutrition diagnosis coded than patients with malnutrition and a BMI in the normal or underweight categories [33]. The limitations of available tools should be recognized when implementing routine screening and assessment of these patients. A summary of the possible uses and limitations of selected tools, including the Malnutrition Screening Tool (MST), the Malnutrition Universal Screening Tool (MUST), the modified NUTrition Risk in the Critically ill (mNUTRIC) score, Nutrition-Focused Physical Exam (NFPE) and Subjective Global Assessment, is presented in Table 2 [24, –39].

**Table 2:** Selected malnutrition screening and assessment tools and limitations to their application in patients with obesity

Screening	Attributes/benefits	Limitations
Malnutrition screening tool (MST)	Risk is calculated based on whether the patient lost weight without trying, the amount of weight loss, and poor appetite, so it is quick and straightforward to administer	Does not consider body composition changes, only recent weight loss
	Does not rely on low BMI as an indicator	Requires self-reported data from the patient, so not ideal for those who are unable to communicate
	Endorsed by AND. Received a “good/strong” rating compared with other tools that only received a fair evidence grade; recommended regardless of patient age or practice setting [34]	
Malnutrition universal screening tool (MUST)	Risk is calculated based on BMI, unplanned weight loss, and acute disease effect, therefore quick and straightforward	Relies on BMI without considering body composition changes; not sensitive for patients with obesity [35]
		Weight loss is described as “unplanned;” may have been “planned” by patient through unhealthy practices or secondary to acute illness that may not be captured
		Requires patient-reported data; not ideal for those who are unable to communicate
		Not specific to critical illness; lower prognostic value in predicting 28-day mortality than the modified NUTrition Risk in the Critically ill (mNUTRIC) score [36]
Modified NUTrition risk in the critically ill (mNUTRIC) score	Risk based on age, comorbidities, Acute Physiology and Chronic Health Evaluation II score, Sequential Organ Failure Assessment score, days in hospital before ICU admission; interleukin-6 may or may not be added	Intended to identify patients who would most benefit from nutrition support, not necessarily those with malnutrition
	Does not rely on low BMI as an indicator	More difficult to calculate
	Does not require self-reported data from the patient	
	Familiar in a range of practice settings	
	Endorsed by 2016 ASPEN/SCCM guidelines [23]	
Nutrition risk score 2002	Risk based on weight loss or low BMI, reduced food intake, and severity of disease, therefore quick and straightforward	Intended to identify patients who would most benefit from nutrition therapy, not necessarily those with malnutrition
	Endorsed by 2016 ASPEN/SCCM guidelines [24]	Relies on BMI and weight change without considering body composition changes
**Assessment**		
Nutrition-focused physical exam (NFPE)	AND and ASPEN recommend six criteria be assessed to identify malnutrition: weight loss, reduction in dietary intake, subcutaneous fat loss, loss of muscle mass, fluid accumulation, and declining functional status. If two or more are present, a nutrition diagnosis of malnutrition is supported [37]	Requires training/expertise
	Does not rely on low BMI as an indicator	May be more challenging if a baseline NFPE is not available to compare to
	AND and ASPEN endorse the NFPE to collect certain criteria (loss of fat and muscle mass and edema) for the nutrition diagnosis of malnutrition [37]	NFPE may be more difficult in patients with generalized edema
	The NFPE may also be modified to assess for clinical signs of micronutrient deficiencies	Excess adiposity may present barriers to accurately assessing muscle or fat mass loss
Subjective global assessment (SGA) and patient-generated SGA	Assessment criteria include weight, weight history, food intake, symptoms that may impact dietary intake, activities/function, comorbidities, metabolic factors, and physical exam	Requires training because as indicated, it is subjective
	Does not rely on low BMI as an indicator	May rely on patient-reported data; not ideal for those who are unable to communicate
	Based on clinical reasoning; has been used in various settings, including ICU, with good predictivity for complications and mortality	Excess adiposity may present barriers to accurately assessing muscle or fat mass
Global Leadership Initiative on Malnutrition (GLIM)	Assessment criteria include factors that may contribute to etiology of malnutrition (inflammation and reduced dietary intake or assimilation) and phenotypic criteria (non-volitional weight loss, low BMI, and reduced muscle mass) [38]	Low BMI has been reported to be one of the most often used criterion [39]; therefore, utility in patients with obesity is unclear
	Muscle mass may be assessed through “dual-energy absorptiometry (DXA) or corresponding standards using other body composition methods like bioelectrical impedance analysis (BIA), CT, or MRI. When not available or by regional preference, physical examination or standard anthropometric measures like mid-arm muscle or calf circumferences may be used.” [38]	Weight loss is described as “non-volitional;” therefore, weight loss that may have been “volitional” by the patient but achieved through unhealthy practices or secondary to acute illness may not be captured
		The preferred measurements of muscle mass–DXA, CT, and MRI–may not be feasible or available for many ICU patients
Anthropometry	Inexpensive	Influenced by edema and assessor technique
	Easily conducted in clinical setting	Patient positioning may make accurate assessment challenging
		Cutoff values do not consider obesity
		Unable to differentiate adipose from muscle tissue
Bioimpedance	Easily conducted in clinical setting	Influenced by hydration
		Limited in assessing those with obesity
Ultrasonography	Easily conducted in clinical setting, assesses muscle mass	Requires expertise and training
		May be difficult to assess muscle according to the amount of adiposity
Tomography and magnetic resonance	Provides detailed information on muscle mass	Expensive
		Inconvenient methods
		May not be feasible or available for many ICU patients
Biochemical parameters (albumin, transthyretin)	Commonly available	Influenced by hydration and inflammation and therefore, not an accurate measure of nutrition status in many ICU patients
	Inexpensive	
Dynamometry	Assesses muscle functionality	Not feasible in many ICU patients due to sedation and neuromuscular blockade
		May be less informative if a baseline measurement is not available to compare to

*AND* Academy of Nutrition and Dietetics, *ASPEN* American Society for Parenteral and Enteral Nutrition, *BMI* body mass index, *CT* computed tomography, *ICU* intensive care unit, *MRI* magnetic resonance imaging

Many screening tools consider low BMI when calculating nutritional risk. It is essential to understand that BMI as a sole indicator is not directly predictive of poor outcomes, especially among critically ill patients with obesity [40]. Therefore, tools such as MUST, which include BMI as a marker of risk, may be of limited value [22, 33]. For example, in one retrospective study of mechanically ventilated patients admitted for more than 7 days to the ICU, refeeding hypophosphatemia/syndrome, which is commonly seen in malnourished individuals, occurred in almost 37% of subjects, but baseline characteristics, including BMI, were poor predictors of risk [41].

The Nutrition-Focused Physical Exam (NFPE) can be used to assess nutritional status in patients found to be at risk of potential macro- or micronutrient deficits, including in patients with obesity. During the NFPE, the clinician 1) assesses the patient’s general appearance and compares initial impressions with available patient data from medical records or other sources; 2) evaluates the patient’s current body habitus and compares usual BMI and weight changes with these findings; and 3) performs a hands-on assessment of subcutaneous fat mass, muscle mass, and edema and evaluates skin, hair, nails, and the oral cavity to note clinical signs of micronutrient deficiencies or excesses [37, 42–44]. Risk factors for micronutrient deficiencies in patients with obesity may include history of bariatric surgery, underlying fatty liver disease, and the use of certain medications for common comorbidities such as hypertension [45]. Finally, this assessment may also include measures to identify signs of poorly managed chronic conditions such as dyspnea and acanthosis nigricans. Although the NFPE may be routine in some settings, excess adiposity may present barriers to accurately assessing muscle wasting or fat loss.

The subjective global assessment (SGA) tool, validated for different populations including critical care patients, focuses on essential clinical variables that can be obtained from the patient or family members of those who cannot provide their nutritional history. The SGA is a low-cost, noninvasive tool based on a patient’s medical history and physical examination. Due to these characteristics, it has become a commonly used tool for hospitalized patients in various clinical situations. This instrument has also been shown to be a good prognostic assessment tool. One cross-sectional study found that SGA was a reliable tool for identifying malnutrition in patients with overweight and obesity requiring mechanical ventilation [46]. Another study indicated that critically ill patients, including those with obesity and diagnosed as malnourished by SGA, presented with higher ICU readmissions as well as increased mortality [47].

Other assessment instruments like anthropometry and bioimpedance are influenced not only by the hydration status of the patients but also by the cutoffs for normality that may not be appropriate for patients with obesity. Ultrasonography, which can be done at the bedside, seemed to be a promising assessment of the muscle, which is a compartment of the nutritional status, but it requires training and may be technically challenging in patients with obesity. Furthermore, sophisticated tools such as tomography and magnetic resonance may not be routinely available unless performed for diagnosis of the current condition, but even if so, they require software and expertise to interpret results. Muscle function associated with the nutritional status is also difficult to assess once patients are under sedation or neuromuscular blocker therapy. Biochemical parameters like albumin and transthyretin are influenced by the inflammatory status of the patient and, therefore, not useful indicators of nutritional status [48]. More recently, the Global Leadership Initiative on Malnutrition (GLIM) has been proposed as a framework for the diagnosis of the nutritional status; however, it has not been adequately validated to advocate its use in patients with obesity [49, 50].

### Current guidelines for screening and assessment

Notably, the 2019 ESPEN guidelines did not recommend any specific tool to be used in critically ill patients and instead stated that “Every critically ill patient staying for more than 48 h in the ICU should be considered at risk for malnutrition [23].” Meanwhile, the 2016 American Society of Parenteral and Enteral Nutrition (ASPEN) and the Society of Critical Care Medicine, as well as the updated ASPEN guidelines do not address the topic [24, 26]. Neither guideline specifically highlights whether or how screening and assessment practices should differ for patients with obesity.

### Clinical considerations

Available malnutrition screening and assessment tools were not specifically developed for patients with obesity. Overall, assessments should consider the possibility of underlying malnutrition, signs and symptoms of micronutrient deficiencies, and poor muscle quality despite the presence obesity should be considered [45, 51]. Therefore, a good clinical assessment along with an adequate physical examination is required for proper nutritional diagnosis. It is also important to monitor patients for refeeding syndrome, as they may have risk factors unrelated to BMI.

## Estimating energy needs for patients with obesity

There is no current consensus among clinicians and a lack of definitive research on how best to estimate energy needs for patients with obesity in critical care settings. Frequently, measured energy expenditure in critically ill patients with obesity is higher than patients of lower BMI, and energy expenditure tends to increase with increasing BMI [52]. Patients with obesity expend more energy due to higher weight burden. Current evidence supports that increased daily activity increases overall energy expenditure compared to a sedentary lifestyle for those with obesity [53]. Thus, an individualized approach is needed [54]. Indirect calorimetry is the most accurate measure of energy needs and is the reference standard for critically ill patients. There are limitations to its use in critical care; however, recent technological advances allow for broader use of indirect calorimetry in the intensive care unit [55, 56]. Predictive equations are also available, including the Penn State and Modified Penn State equations which were developed in critically ill cohorts, including patients with a wide range of BMIs [57–59]. In general, predictive equations demonstrate a low to moderate level of performance but may still be useful as a starting point in some clinical settings due to the inherent time and logistic limitations of routine indirect calorimetry [55, 56]. A recent analysis compared measured energy expenditure to weight-based equations recommended in the 2016 ASPEN/SCCM guidelines. They reported clinically significant variations and concluded that “a one-size-fits-all approach to estimation of energy expenditure at a single time point is likely to be inappropriate in critical illness [24, 52].” Finally, there is no consensus on whether actual, ideal, or adjusted body weight should be used to calculate energy needs when using these equations, especially for critically ill patients with obesity [23–25, 60].

### Current guidelines on energy provision

The 2013 ASPEN obesity guidelines suggest that if indirect calorimetry is unavailable, energy requirements should be based on the Penn State University 2010 predictive equation, or the modified Penn State equation if the patient is over the age of 60 years [25]. The 2016 ASPEN/SCCM guidelines echoed the preference for indirect calorimetry and stated that, if unavailable, a simple weight-based equation be used [24]. Specifically, these guidelines suggest that enteral nutrition be provided within 24–48 h of admission to patients with obesity and suggest an energy provision of 11–14 kcal/kg actual body weight for individuals with a BMI of 30–50 kg/m^2^ and 22–25 kcal/kg ideal body weight for individuals with a BMI greater than 50 kg/m^2^ [24]. In 2019, ESPEN released guidelines suggesting clinicians add 20%–25% of the difference in actual body weight minus ideal body weight added to ideal body weight when calculating energy requirements for patients with obesity [23] as estimating caloric intake on ideal body weight may underestimate energy expenditure for those with obesity. It should be noted that all available guidelines are based on a low quality of evidence, and none contemplate which ideal body weight calculation to use. The current ESPEN guidelines, for instance, discuss three different methods for calculating ideal body weight [23], with no consensus for clinicians on which might be optimal for their patients.

In the absence of consistent guidelines to estimate energy needs for these patients, clinicians should use an individualized approach that considers current guidelines, the amount of metabolically active tissue the patient may have (which will influence the degree of hypermetabolism) and the need to respond to changing nutritional requirements and metabolic aberrations throughout the course of care. Patients should be closely monitored, and nutrition regimens should be adjusted accordingly for outcomes such as hyperglycemia, hypercapnia, and other metabolic disturbances.

### Clinical considerations

It is important to understand the limitations of available tools when calculating energy needs and their low applicability to patients with obesity. If utilized, predictive equations should only be one aspect of a nutrition assessment for these complex patients and limitations should be recognized. Clinical staff should review individual nutritional requirements and consider factors that affect energy needs on a regular basis such as changes in clinical status including fever and infection, major postoperative procedures, and increased mobility and physical activity. This is necessary as patients’ needs can frequently and rapidly change over the course of critical illness. Finally, regardless of BMI and the determined energy target, it is important to recognize that hospitalized patients, especially those in the ICU, frequently do not consume or receive the amount of nutrition prescribed [52, 61]. Ultimately, the nutritional management of critically ill patients with obesity should be undertaken with the intention of reducing net protein catabolism without concurrent feeding complications and worsening of physical function and clinical outcomes.

## Protein requirements in the ICU

Factors such as advanced age, immobility, inflammation, insulin resistance, and medications may increase the protein requirements of critically ill patients [24, 62]. These factors, along with chronic low protein provision in many patients with obesity, may contribute to the loss of lean body mass and the development of sarcopenic obesity, as well as ICU-acquired weakness. Sarcopenia and cachexia are both more common than previously recognized in critically ill patients with obesity [63, 64]. Sarcopenic obesity is estimated to impact 11% of community-dwelling older adults and 16% of older adults in the hospital setting, suggesting that additional screening for earlier diagnosis is needed to provide effective intervention and improve outcomes [65]. Meanwhile, there is no consensus on protein requirements in patients with obesity, leaving clinicians to depend on limited evidence and current guidelines. Ultimately, muscle mass loss may still occur in critically ill patients despite the delivery of what may be determined as adequate protein provision.

### Current guidelines for protein provision

To address protein requirements, the ESPEN 2019 guidelines suggest that protein delivery should be guided by urinary nitrogen losses or lean body mass assessments. Use of urinary nitrogen losses to estimate nitrogen balance can be challenging as it requires an accurate 24-h record of nutritional intake and urine collection. It is difficult to obtain a precise 24-h urine collection especially for those without an indwelling urinary catheter. Intestinal drainages and stool losses cannot be measured and requires an assumption of estimated integumentary and urinary non-urea nitrogen losses. Finally, nitrogen balance is reflective of the net difference between nitrogen intake and nitrogen losses. It is limited in that it cannot detect whether anabolism or catabolism is being influenced by the nutrition therapy [66]. The ESPEN 2019 guidelines recommend assessment of lean body assessment via ultrasound, computed tomography scan, magnetic resonance imaging, dual-energy absorptiometry, or bioelectrical impedance [23].

If these tools are unavailable, the progressive delivery of 1.3 g/kg of protein per day based on adjusted body weight is recommended [23]. Likewise, the 2013 ASPEN guidelines suggest beginning with 1.2 g protein/kg actual weight, or 2–2.5 g/kg ideal body weight of protein for patients who are overweight or have obesity, with adjustments based on results of nitrogen balance studies [25]. The 2016 ASPEN/SCCM obesity guidelines suggest 2 g protein/kg ideal body weight for patients with BMI of 30–40 kg/m^2^ and up to 2.5 g/kg ideal body weight for those with a BMI ≥ 40 kg/m^2^ [24].

Muscle protein synthesis following protein administration is blunted in critical illness [67]. However, indirect evidence, using nitrogen balance determinations, indicates that this anabolic resistance can be mediated by increasing protein intake in critically ill trauma patients with or without obesity [26, 27, 68]. Current guidelines reflect evidence that higher levels of protein delivery have been associated with positive patient outcomes in retrospective and prospective observational studies and small randomized controlled trials, which have not been definitively determined in adequately sized randomized controlled trials. One prospective cohort study in mechanically ventilated critically ill patients (mean BMI: 26 ± 6 kg/m^2^) found that optimal nutritional therapy, including adequate protein and energy delivery, was associated with a 50% decrease in 28-day mortality. Adequate energy delivery without meeting protein targets, however, was not associated with mortality risk [69]. In another study of 1,171 critically ill patients with a mean BMI of 28 kg/m^2^, increased protein intake was associated with a modest reduction in mortality risk [70]. Thus, higher protein delivery, which may be achieved with the use of high protein formula or protein modular supplements, may be preferred. For instance, a double-blind, randomized trial found that a very high-protein enteral formula (32% kcals from protein) compared to a conventional, standard high-protein content formula (20% kcals from protein) demonstrated the ability to deliver adequate protein requirements without increased energy intake in critically ill patients who were overweight [71].

### Clinical considerations

A personalized approach to nutrition is key to delivering adequate protein while reducing the risk of excessive energy provision for critically ill patients with obesity to reduce morbidity and mortality risks [72]. A practical approach to developing this type of nutritional regimen would be the use of high protein, low calorie enteral formulas with the addition of bolus protein supplements if necessary for those require enteral nutrition therapy. For parenteral nutrition, a low dextrose, high amino acid content formula would be prescribed. However, other aspects must be taken into consideration, such as renal and hepatic function. As previously discussed, frequent reassessment of clinical status and nutritional needs is vital in these vulnerable patients.

## Nutrition strategies for patients with obesity in the ICU

Considering previously discussed nutrition requirements in the critical care setting for all patients, including those with obesity, and understanding the importance of avoiding overfeeding, a tailored approach with fewer calories and more protein seems reasonable. Although data are currently limited and only available from small study populations, hypocaloric, high-protein nutrition therapy has been found to decrease net protein catabolism in hospitalized and critically ill patients with obesity [26, 73–76]. One such study indicated improved clinical outcomes with hypocaloric high-protein feeding compared to eucaloric feeding [73]; however, other studies [74, 75] indicated no difference. More recently, the augmented versus routine approach to giving energy trial (TARGET) evaluated energy provision of 1.0 kcal/mL versus 1.5 kcal/mL in 3957 patients in the setting of critical illness. There was no significant difference in all-cause mortality at 90 days, infectious complications, or adverse events in the overall study group or in the subgroup with obesity [77]. However, interpretation of these data is tempered, as both groups potentially may have received inadequate protein intakes (~ 1.1 g/kg ideal body weight or ~ 0.8 g/kg actual weight daily).

Anabolic resistance, as assessed by nitrogen balance or whole-body protein dynamics using isotopes, associated with aging can be overcome with sufficient protein intake during critical illness [27] even during hypocaloric energy intake for critically ill older patients with obesity [28]. The extent of obesity also appears to influence the amount of protein required as those patients with class III obesity needed more protein to achieve an equivalent nitrogen balance compared to those with class I and II obesity [78].

### Current guidelines

Based on this and other evidence, 2016 guidelines from ASPEN/SCCM suggest fewer calories and a high-protein diet for patients with obesity [24, 25]. Specifically, based on expert consensus, the guidelines state, “if available, an enteral formula with low caloric density and a reduced NPC:N [nonprotein calorie:nitrogen ratio] be used in the adult obese ICU patient [24].”

### Clinical considerations

Like other aspects of nutritional management in critically ill patients with obesity, clinicians should consider an individualized approach to nutritional management that achieves a higher protein intake without overfeeding energy. While fewer calories are appropriate, a high-protein nutrition regimen may be preferred for many critically ill patients with obesity. While debate ensues regarding the duration and ideal protein delivery during hypocaloric feeding, a target of 2 to 2.5 g/kg ideal body weight per day is considered reasonable in critically ill patients with obesity. While all calculations of ideal body weight have certain limitations, the Hamwi formula may be used in this setting [79]. Monitoring patients for adequate protein is another area of controversy and challenges, especially if a 24-h urine collection is required for nitrogen balance analysis. Limited data are available on proper interpretation of nitrogen balance results, and it is still unclear how these findings correlate with clinical outcomes.

## The role of immunonutrition and fiber in critically Ill patients with obesity

The excessive visceral fat tissue and adipocyte hypertrophy seen in patients with obesity contributes to a pro-inflammatory environment, including higher levels of hormones like leptin that disrupt T cell function, which results in a suppressed immune response to infection [80]. Immune function may be influenced by nutritional factors such as vitamin D and arginine status, both of which have been reported to be reduced in non-ICU patients with obesity [81–84]. Beyond arginine and vitamin D, inflammation may be modulated by other dietary factors such as omega-3 fatty acids and fiber; therefore, the use of these immune-modulating nutrients (also known as immunonutrition) has been considered in critically ill patients with obesity who are hemodynamically stable.

The enteric microbiome is integral to the development and function of innate/adaptive immune systems through microbial metabolites and is likewise impacted by leptin and other hormones that are motivated by a pro-inflammatory environment [85–87]. These same pathways are hypothesized to play an important role in the initial development of obesity as well [88, 89]. Nutrition, including the route of nutrition (enteral versus parenteral) [90, 91] and nutrition components such as soluble fiber [92, 93], have been shown to alter the gut microbiota. The microbiome is, therefore, being investigated as a target to address inflammation and obesity, with immunonutrition a potential modulating factor in this setting.

### Current guidelines

Based on expert opinion or very low to moderate quality of evidence, the 2016 ASPEN/SCCM guidelines suggest immune-modulating formulas with components such as arginine and fish oil in the surgical intensive care unit, for severe trauma patients, and for those with traumatic brain injury [24]. It is not clear if immunonutrition could benefit critically ill patients with obesity who do not have these conditions. The 2016 ASPEN/SCCM guidelines state, “while an exaggerated immune response in obese patients implicates potential benefit from immunomodulating formulas, lack of outcome data precludes a recommendation at this time [24].” Regarding soluble fiber, the 2016 ASPEN/SCCM guidelines suggest “a fermentable soluble fiber additive (e.g., fructooligosaccharides [FOSs], inulin) be considered for routine use in all hemodynamically stable MICU [medical intensive care unit]/SICU [surgical intensive care unit] patients placed on a standard enteral formulation [24].” This guidance is for the general ICU patient and the guidelines do not mention whether fiber provision should differ for patients with obesity.

### Clinical considerations

Although immunonutrition may modulate inflammation in critically ill patients with obesity, its clinical relevance has not yet been fully elucidated. Until further evidence is available, clinicians should use their clinical judgment and evaluate individual patient situations when considering immunonutrition adjuncts in critically ill patients with obesity.

## Educational opportunities to support patients

Patients with obesity are complex and present in the critical care setting with a high degree of variability in baseline risk for poor outcomes [94–96]. Patient size does not necessarily predict individual risk. This message and the need for specialized, individualized nutrition therapy requires ongoing educational efforts for all clinical staff involved in the management of these complex patients.

Critical care staff, including physicians, nurses, dietitians, and pharmacists involved in the clinical care of patients with obesity, require adequate knowledge to provide optimal nutrition that maintains physical function and reduces risk of poor outcomes [94–96]. In addition, clinicians should consider the unique logistic and equipment needs for this population, as well as the use of strategies such as proper patient positioning to reduce pressure injury risk [97], maintain the airway, and prevent aspiration due to unique anatomical challenges [95, 96]. Repositioning and ambulation may be more difficult in patients with obesity, however, are important for optimal care and may have important impacts on the nutrition status and needs of the patient [98]. For example, inadequate repositioning may increase risk for pressure injury development which can alter the patient’s requirement for calories, protein, and micronutrients necessary for wound healing [99]. Early mobilization has been encouraged for functional recovery and maintenance of muscle health; therefore, nutrition needs may be impacted during and following discharge if not routinely performed [100].

Educational efforts should highlight that weight stigma and bias may affect the delivery of care [94, 95, 101]. To treat the whole person, staff should be encouraged to use person-first language and recognize the possibility that patients with obesity may have experienced stigma or negative experiences with healthcare in the past. Finally, in addition to using clinical judgment, clinicians should remain informed of updated guidelines from nutritional societies and evolving research on the topics discussed in this review.

## Conclusion

Patients with obesity and critical illness present with several clinical challenges due to the possibility of underlying comorbidities, systemic inflammation, and a lack of evidence to routinely guide nutritional management interventions. Considering the challenges in providing optimal care for patients with obesity and the lack of robust evidence, this advisory board’s consensus opinion rests on the importance of individual care with the principal of first, do no harm. Overall, current nutritional strategies for these patients should be undertaken with a focus on individualized care that considers the whole person, including the possibility of chronic stigma that could impact the delivery of effective care. Additional research should focus on the applicability of current guidelines and evidence for nutrition therapy in populations with obesity. Future studies should also be undertaken to determine the potential for strategies that better address underlying inflammation and other chronic risks seen in patients with obesity, especially in the setting of critical illness.

## Data Availability

Not applicable

## Abbreviations

AND: Academy of Nutrition and Dietetics
ASPEN: American Society for Parenteral and Enteral Nutrition
BIA: Bioelectrical impedance analysis
BMI: Body mass index
CT: Computed tomography
DXA: Dual-energy absorptiometry
ESPEN: European Society for Clinical Nutrition and Metabolism
FOS: Fructooligosaccharide
GLIM: Global Leadership Initiative on Malnutrition
IC: Indirect calorimetry
ICU: Intensive care unit
IMN: Immunonutrition
MICU: Medical intensive care unit
mNUTRIC: Modified nutrition risk in the critically ill
MRI: Magnetic resonance imaging
MST: Malnutrition screening tool
MUST: Malnutrition universal screening tool
NFPE: Nutrition-focused physical exam
SCCM: Society of Critical Care Medicine
SGA: Subjective global assessment
SICU: Surgical intensive care unit
TARGET: The augmented versus routine approach to giving energy trial
TBI: Traumatic brain injury
WHO: World Health Organization
