# Correction: Obesity and critical care nutrition: current practice gaps and directions for future research

**DOI:** 10.1186/s13054-023-04456-z

**Published:** 2023-05-08

**Authors:** Roland N. Dickerson, Laura Andromalos, J. Christian Brown, Maria Isabel T. D. Correia, Wanda Pritts, Emma J. Ridley, Katie N. Robinson, Martin D. Rosenthal, Arthur R. H. van Zanten

**Affiliations:** 1 Department of Clinical Pharmacy and Translational Science, University of Tennessee Health Science Center, 881 Madison Avenue, Suite 345, Memphis, TN 38163 USA; 2 Clinical Nutrition Manager, Sodexo, Minneapolis, MN USA; 3 Digestive Disease and Surgery Institute, Cleveland Clinic, Cleveland, OH USA; 4 Department of Surgery, Federal University of Minas Gerais, Belo Horizonte, Brazil; 5 Placenta-Linda Hospital, 1301 Rose Dr, Placentia, CA USA; 6 Australian and New Zealand Intensive Care Research Centre, School of Public Health and Preventive Medicine, Monash University, Melbourne, VIC Australia; 7 Scientific and Medical Affairs, Abbott Nutrition, 2900 Easton Square Pl, Columbus, OH USA; 8 Acute Care Surgery Team, University of Florida College of Medicine, Gainesville, FL USA; 9 Chair, Department of Intensive Care Medicine & Research, Gelderse Vallei Hospital, Willy Brandtlaan 10, 6716 RP Ede, The Netherlands; 10 Division of Human Nutrition and Health, Chair Group Nutritional Biology, Wageningen University & Research, HELIX (Building 124), Stippeneng 4, 6708 WE Wageningen, The Netherlands

**Correction: Critical Care (2022) 26:283** **https://doi.org/10.1186/s13054-022-04148-0**

Following publication of the original article [1], the authors reported an error in the Competing Interests section.

The Competing Interests section currently reads:

**Competing Interests**

The authors declare no competing interests.

The Competing Interests section should read:

**Competing Interests**

Katie N. Robinson works and owns stock in Abbott Nutrition.

The Competing Interests section has been updated above and the original article [1] has been corrected.
